# Tamoxifen synergizes with 4-(E)-{(4-hydroxyphenylimino)-methylbenzene, 1,2-diol} and 4-(E)-{(p-tolylimino)-methylbenzene-1,2-diol}, novel azaresveratrol analogs, in inhibiting the proliferation of breast cancer cells

**DOI:** 10.18632/oncotarget.10106

**Published:** 2016-06-16

**Authors:** Amruta Ronghe, Anwesha Chatterjee, Nimee K. Bhat, Subhash Padhye, Hari K. Bhat

**Affiliations:** ^1^ Division of Pharmacology and Toxicology, School of Pharmacy, University of Missouri-Kansas City, Kansas City, Missouri, USA; ^2^ Interdisciplinary Science and Technology Research Academy, Abeda Inamdar Senior College, Department of Chemistry, University of Pune, Pune, India

**Keywords:** breast cancer, tamoxifen, resveratrol analogs, synergistic inhibition, combination therapy

## Abstract

We have recently shown that 4-(E)-{(4-hydroxyphenylimino)-methylbenzene, 1,2-diol} (HPIMBD) and 4-(E)-{(p-tolylimino)-methylbenzene-1,2-diol} (TIMBD), novel analogs of resveratrol (Res), selectively inhibited the proliferation of breast cancer cells. In the current study, we tested HPIMBD and TIMBD individually in combination with tamoxifen (Tam) for inhibition of growth of breast cancer cells. Tamoxifen was first tested on non-neoplastic breast epithelial cell lines and its dose that does not inhibit their growth was determined. A combination of this low dose of Tam with either of the Res analogs HPIMBD or TIMBD, resulted in synergistic inhibition of proliferation of breast cancer cells. Both estrogen receptor (ER)-positive and negative breast cancer cell lines responded to the combination. The combination resulted in a substantial decrease in IC_50_ values of Res analogs in all breast cancer cell lines tested. Mechanistic studies showed a synergistic increase in apoptosis and autophagy genes (beclin-1 and LC3BII/I) with the combination in ER-negative MDA-MB-231 cells. In ER-positive MCF-7 and T47D cells, the mechanism of synergy was found to be inhibition of expression of ERα and oncogene c-Myc. The combination treatment had a synergistic effect in inhibiting the colony forming and spheroid forming ability of cancer cells. Taken together, our findings indicate that a combination of Tam and Res analogs HPIMBD or TIMBD represents a novel approach to enhancing the use of Tam in therapy for breast cancers. Considering the urgent need for novel therapeutic strategies to treat ER-negative breast cancers and overcoming resistance in ER-positive cancers, this combinatorial approach is worthy of continued investigation.

## INTRODUCTION

Breast cancer is the second leading cause of cancer deaths among women in the United States [1–3]. Prolonged exposure to estrogens is considered as a major risk factor in the development of breast cancers [4–7]. In 2001, steroidal estrogens were added to the list of known human carcinogens [8]. Both estrogen receptor-mediated and estrogen metabolism-mediated pathways have been implicated as molecular mechanisms of estrogen-induced breast carcinogenesis [9–11]. Estrogens are known to act through their binding to estrogen receptors (ERs) α and β, leading to their activation [12, 13]. Activation of ERα leads to cellular proliferation by induction of cellular oncogenes like c-myc and cell cycle proteins like cyclin D1, that result in progression of cancer [12, 13]. More than 70 % of clinical breast cancers overexpress ERα and are hormone responsive [14]. Currently, the expression of ERα is considered as a prognostic marker to predict response to anti-hormonal therapy [15]. Tamoxifen is widely used in the treatment of ER positive breast cancers and has also been approved as a chemopreventive agent for women with high risk for breast cancer [16, 17]. Tamoxifen is classified as a selective estrogen receptor modulator (SERM) that acts as an antagonist of ERα in the breast [18]. Tamoxifen mainly inhibits the proliferation of ER-positive breast cancer cells by competing with estrogens' binding to ERα [18, 19]. Tamoxifen has also been shown to mediate apoptosis in an ERα-independent manner by regulation of several signaling targets such as protein kinase C, transforming growth factor-β, calmodulin, mitogen-activated protein kinases (MAPK) p38 and c-Jun terminal kinases [20]. Apart from inducing ERα-independent apoptosis, Tam is also known to induce autophagy, a process for recycling cellular components during stress/starvation conditions, in breast cancer cells [21, 22]. However, despite the multiple molecular targets, Tam therapy remains primarily tumoristatic and long-term Tam treatment leads to serious adverse effects such as development of endometrial cancers [23, 24, 25]. Another factor that limits the efficacy of Tam is the development of endocrine resistance [26]. Studies suggest that endocrine-resistance may account for up to one-quarter of all breast cancers [26]. Several mechanisms such as loss of ERα expression, mutations in ERα and activation of other survival signaling pathways have been implicated in the development of Tam resistance [27]. In order to overcome the adverse effects of Tam, combination therapies with other synthetic and natural agents have been suggested [28–31]. Combination therapy not only reduces the effective dose of Tam, leading to decreased toxicity but also has the potential of synergistic inhibition and re-sensitization of resistant cancer cells [28–31]. Although combination with synthetic agents does improve the therapeutic outcomes and life-expectancy, their toxicities also add up [32]. Thus, combination therapies with agents of natural origin have been suggested [29–31].

Phytoestrogens from plants have been regarded as important untapped sources of potential anticancer molecules [33, 34]. Evidence suggests that early exposure to high phytoestrogen containing diets may reduce the risk of breast cancer in women [33, 34]. Resveratrol (Res, 3,5,4′-trihydroxystilbene) is a naturally occurring phytoestrogen found abundantly in red grapes, berries and nuts that has shown potent anti-cancer activity [35]. Resveratrol has been shown to inhibit the proliferation of breast cancer cells in *in vitro* and xenograft studies [36]. Resveratrol induces apoptosis and cell cycle arrest in cancer cells, which are its primary mechanisms of cancer inhibition [36]. Resveratrol has sensitized resistant breast cancer cells to Tam in combination studies by induction of transforming growth factor-β signaling pathways [37]. However, extensive metabolism and poor oral bioavailability of less than 1% have limited the use of Res in clinical studies [38]. To improve the anti-cancer potential of Res we have recently synthesized five azaresveratrol analogs resembling the basic skeleton of Res and having additional pharmacophoric groups [39]. Structurally, the 3,4-dihydroxy substituents on the A ring of Res have been maintained and those on the C-4 position in the B ring have been varied along with inclusion of the aza functionality in the conjugated system [39]. These novel azaresveratrol analogs were characterized, purified and screened for their anti-cancer activities against breast cancer cell lines [39]. Two analogs, 4-(E)-{(4-hydroxyphenylimino)-methylbenzene, 1, 2-diol} (HPIMBD) and 4-(E)-{(p-tolylimino)-methylbenzene-1,2-diol} (TIMBD) (please note that HPIMBD and TIMBD are referred to as compounds 3e and 3b in reference # 39, and HPIMBD and TIMBD are their chemical names according to IUPAC nomenclature) showed better potency than Res in inhibiting the proliferation of breast cancer cell lines following cell viability assays [39]. Also, HPIMBD and TIMBD did not have any effect on the proliferation of normal breast epithelial cells up to a concentration of 50 μM, suggesting their safety towards normal breast epithelial cells and selectivity for cancer cells [39]. It was also observed that both HPIMBD and TIMBD induced the protein expression levels of beclin-1 protein, an accepted biomarker for the induction of autophagy, suggesting that autophagy induction may be one of the pathways activated by novel Res analogs [39].

In the present study, we have performed combination treatments with Tam and Res analogs HPIMBD and TIMBD in breast cancer cell lines. We demonstrate that a combination of low dose Tam with HPIMBD or TIMBD does not inhibit the growth of non-neoplastic breast epithelial cells, suggesting lower toxicity of this combination. The combination, on the other hand, has a synergistic effect in the inhibition of growth of breast cancer cell lines. We demonstrate that the mechanisms of synergistic inhibitory effects in breast cancer cells differ and correlate with their receptor status. In ERα-negative MDA-MB-231 cells, the synergistic effect seems to be mediated by induction of early autophagy and late apoptosis while in ERα-positive MCF-7 and T47D cells, inhibition of proliferation may be mediated by the synergistic inhibition of ERα and c-Myc expression.

## RESULTS

### Low dose Tam in combination with HPIMBD or TIMBD, did not have any effect on the growth of normal breast epithelial cell lines

**Figure 1:**
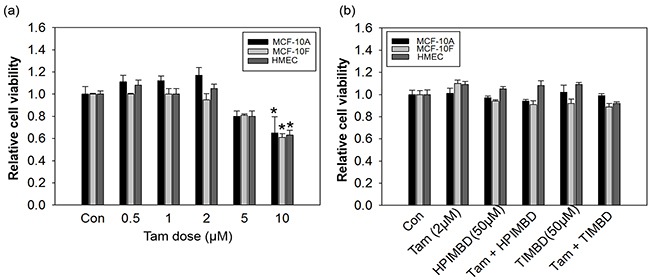
Low dose tamoxifen in combination with resveratrol analogs HPIMBD or TIMBD does not inhibit the growth of non-neoplastic breast epithelial cells **a.** Breast epithelial cell lines MCF-10A, MCF-10F and HMEC were treated with varying doses of tamoxifen ranging from 0.5 to 10 μM for 72 hours and MTT assays were performed. Data are expressed as relative cell viabilities of treatments compared to vehicle (DMSO) control plotted against the dose of treatments. Fold change was determined by dividing the average optical density of treated cells by that of control cells. Each experiment was performed in quadruplicates and the data are expressed as fold change ± SEM relative to control. (*) indicates a P value ≤ 0.05 compared to controls of the same cell type. **b.** Non-neoplastic breast epithelial cell lines MCF-10A, MCF-10F and HMEC were treated with either vehicle or 2 μM Tam, 50 μM HPIMBD or TIMBD or a combination of Tam + HPIMBD or TIMBD for 72 hours and MTT assays were performed. Relative cell viability was determined by dividing the absorbance in treated cells by that in vehicle-only cells. Each experiment was performed in quadruplicates and data are expressed as fold change ± SEM relative to respective vehicle-treated controls. (*) indicates a P value ≤ 0.05 compared to controls of the same cell type.

Non-neoplastic breast epithelial cell lines MCF-10A, MCF-10F and HMEC were initially treated with different doses of Tam ranging from 0.5 - 10μM in concentration and MTT cell survival assays were performed after 72 hours. A dose of 2 μM did not show any effect on the proliferation of non-neoplastic breast epithelial cells (Figure 1a) and thus was chosen for subsequent combination studies with HPIMBD and TIMBD. We have recently shown that HPIMBD and TIMBD did not have any effect on the proliferation of breast epithelial cell line MCF-10A up to a dose of 50 μM [39]. A similar effect was seen after treatment of MCF-10F and HMEC cell lines with 50 μM doses of HPIMBD or TIMBD (Figure 1b). Normal breast epithelial cell lines MCF-10A, MCF-10F and HMEC were then treated with a combination of 2 μM and 50 μM doses of Tam and HPIMBD or TIMBD, respectively. The combination treatments also did not inhibit the growth of the above tested non-neoplastic breast epithelial cells (Figure 1b).

### A combination of Tam with HPIMBD or TIMBD has a synergistic inhibitory effect on the growth of breast cancer cell lines

**Figure 2:**
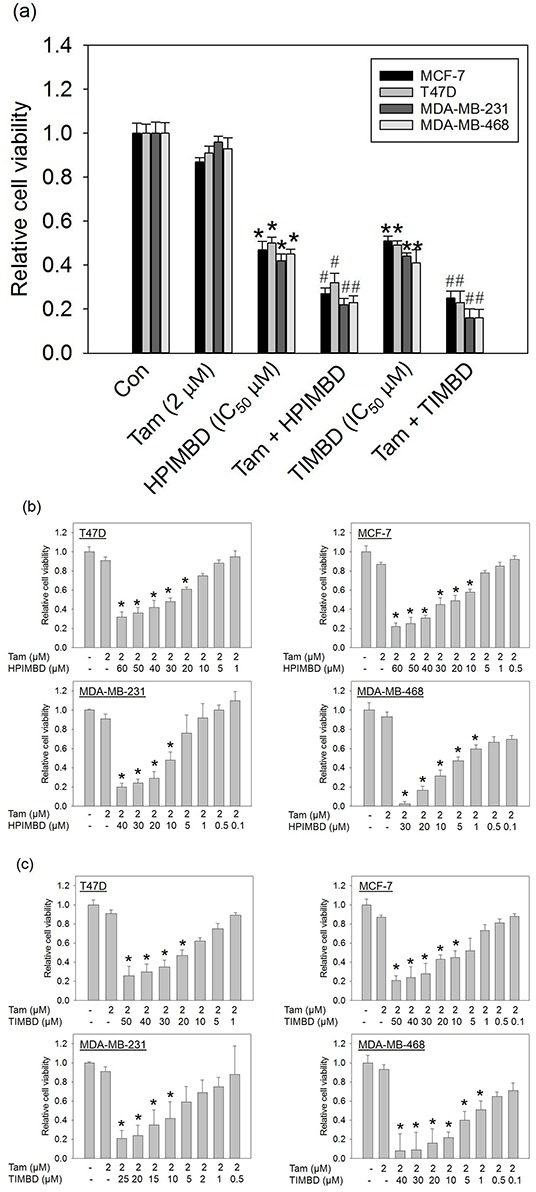
Low dose tamoxifen in combination with HPIMBD or TIMBD synergistically inhibits the growth of breast cancer cells and substancially reduces the IC_50_ values of HPIMBD and TIMBD **a.** Breast cancer cell lines MCF-7, T47D, MDA-MB-231 and MDA-MB-468 were treated with either vehicle or 2 μM Tam, HPIMBD or TIMBD (IC_50_ μM) or a combination of Tam + HPIMBD or TIMBD for 72 hours and MTT assays were performed. Relative cell viability was determined by dividing the absorbance in treated cells by that in vehicle-only cells. Each experiment was performed in quadruplicates and data are expressed as fold change ± SEM relative to respective vehicle-treated controls. (IC_50_ values in μM of HPIMBD in breast cancer cell lines T47D = 44, MCF-7 = 41, MDA-MB-231 = 29, MDA-MB-468 = 5; IC_50_ values in μM of TIMBD in breast cancer cell lines T47D = 32, MCF-7 = 31, MDA-MB-231 = 21, MDA-MB-468 = 4). (*) indicates a P value ≤ 0.05 compared to vehicle controls of the same cell type. (#) indicates a synergistic effect of the combination of Tam + HPIMBD/TIMBD compared to either single drug treatments. **b.** Breast cancer cell lines were treated with a constant low dose of 2 μM Tam in combination with graded doses of HPIMBD for 72 hours and MTT assays were performed. Relative cell viability was determined by dividing the absorbance in treated cells by that in vehicle-only cells and a dose-response curve was plotted. Each experiment was performed in triplicate and data are expressed as fold change ± SD relative to respective vehicle-treated controls. (*) indicates a P value ≤ 0.05 compared to controls of the same cell type. **c.** Breast cancer cell lines were treated with a constant low dose of 2 μM Tam in combination with graded doses of TIMBD for 72 hours and MTT assays were performed. Relative cell viability was determined by dividing the absorbance in treated cells by that in vehicle-only cells and a dose-response curve was plotted. Each experiment was performed in triplicate and data are expressed as fold change ± SD relative to respective vehicle-treated controls. (*) indicates a P value ≤ 0.05 compared to controls of the same cell type.

We have recently shown that HPIMBD and TIMBD have a selective inhibitory effect on the growth of several breast cancer cell lines [39]. In the current study, the combination of Tam with either HPIMBD or TIMBD was tested on breast cancer cell lines. MCF-7, T47D, MDA-MB-231 and MDA-MB-468 breast cancer cells were treated with a combination of Tam and HPIMBD or TIMBD for 72 hours and MTT assays were performed. Tam was used at a dose of 2 μM while HPIMBD and TIMBD were used at their IC_50_ doses in respective cell lines, as determined previously [39]. A combination of Tam with HPIMBD or TIMBD synergistically inhibited the proliferation of breast cancer cell lines compared to either drug treatment alone (Figure 2a). Tamoxifen alone did not have a significant effect on the proliferation of breast cancer cells. HPIMBD and TIMBD treatments alone resulted in about 50% inhibition of cancer cell proliferation (Figure 2a). The combination, however, resulted in about 70-80% inhibition in cellular proliferation which was statistically synergistic (Figure 2a). The % inhibition of proliferation for either treatment alone and in combination were calculated and have been listed in Table 1. Estrogen receptor positive cell lines MCF-7 and T47D showed about 70% inhibition of cell growth on an average while the ER-negative breast cancer cell lines MDA-MB-231 and MDA-MB-468 showed a higher sensitivity to the combination with around ~80% inhibition (Table 1).

**Table 1:** Percent inhibition in breast cancer cell lines following treatments with tamoxifen, HPIMBD, TIMBD and a combination of Tam + HPIMBD or TIMBD

Cell line	Vehicle control (%)	Tamoxifen 2 μM (%)	HPIMBD IC_50_ (μM) (%)	TIMBD IC_50_ (μM) (%)	Tamoxifen + HPIMBD (%)	Tamoxifen + TIMBD (%)
T47D	0 ± 4.1	9 ± 3.1	50* ± 2.7	51* ± 2.1	68# ± 4.3	77# ± 5.2
MCF-7	0 ± 4.5	13 ± 2.0	53* ± 3.7	49* ± 2.1	73# ± 2.7	75# ± 3.3
MDA-MB-231	0 ± 5.1	4 ± 2.8	58* ± 3.1	56* ± 1.6	78# ± 2.9	84# ± 4.1
MDA-MB-468	0 ± 4.8	7 ± 5.0	55* ± 2.3	59* ± 5.9	77# ± 3.1	84# ± 4.0

(*) indicates a P value ≤ 0.05 compared to controls of the same cell type.

(#) indicates a synergistic effect of a combination of Tam + HPIMBD/TIMBD compared to single drug treatments. Breast cancer cell lines T47D, MCF-7, MDA-MB-231 and MDA-MB-468 were treated with either vehicle or 2 μM Tam, HPIMBD or TIMBD (IC_50_ μM) or a combination of Tam + HPIMBD/TIMBD for 72 hours and MTT assays were performed. Relative cell viability was determined by dividing the absorbance in treated cells by that in vehicle-only cells and percent cell viability was calculated from Figure 2a. Percent inhibition of cellular proliferation was calculated by subtracting percent cell viability from 100. Each experiment was performed in quadruplicate and data are expressed as percent inhibition ± SEM relative to respective vehicle-treated controls.

### The combination of Tam with HPIMBD or TIMBD has significantly reduced IC_50_ in breast cancer cells compared to single drug treatments

We next determined the IC_50_ values of inhibition of breast cancer cells by the combination of Tam and Res analogs HPIMBD or TIMBD. Breast cancer cell lines noted above were treated with a constant dose of Tam (2 μM) in combination with graded doses of HPIMBD or TIMBD from 0.1 – 60 μM for a period of 72 hours and MTT assays were performed (Figure 2b and 2c). Dose-response curves were plotted and IC_50_ values were calculated as described in the Materials and Methods section. HPIMBD and TIMBD showed a dose-dependent inhibition of cellular proliferation in combination with Tam (Figure 2b and 2c). HPIMBD and TIMBD in combination with Tam showed IC_50_ values in the range of 10-30 μM for ER-positive and 2-10 μM for ER-negative breast cancer cells (Table 2). TIMBD in combination with Tam showed lower IC_50_ values compared to HPIMBD, suggesting that it is a better analog in inhibition of breast cancer cell growth (Table 2). When compared to either analog treatments alone, combinations of Tam + HPIMBD or TIMBD showed maximum decreases of 68% and 72% in IC_50_ values in MDA-MB-231 and T47D cells, respectively (Table 2).

**Table 2:** IC_50_ values of a combination of tamoxifen + HPIMBD or TIMBD in breast cancer cell lines

Cell line	Tamoxifen + HPIMBD IC_50_ (μM)	Tamoxifen + TIMBD IC_50_ (μM)	% Decrease in IC_50_ from HPIMBD alone	% Decrease in IC_50_ from TIMBD alone
T47D	29.45 ± 2.68	17.29 ± 2.18	34.3	72.7
MCF-7	20.16 ± 2.64	8.89 ± 3.31	50.5	44.9
MDA-MB-231	9.37 ± 1.76	7.56 ± 2.36	68.2	63.6
MDA-MB-468	3.64 ± 0.77	1.77 ± 0.55	26.5	50.0

Breast cancer cell lines T47D, MCF-7, MDA-MB-231 and MDA-MB-468 were treated with a combination of constant dose of Tam with increasing doses of either HPIMBD or TIMBD for 72 hours and MTT assays were performed. Relative cell viability was determined by dividing the absorbance in treated cells by that in vehicle-only cells. Dose-response curves were plotted and IC_50_ values were determined as described in the Materials and Methods section. Each experiment was performed in triplicate and data are expressed as IC_50_ ± SD.

### Tamoxifen in combination with HPIMBD or TIMBD induces late apoptosis in breast cancer cells

**Figure 3:**
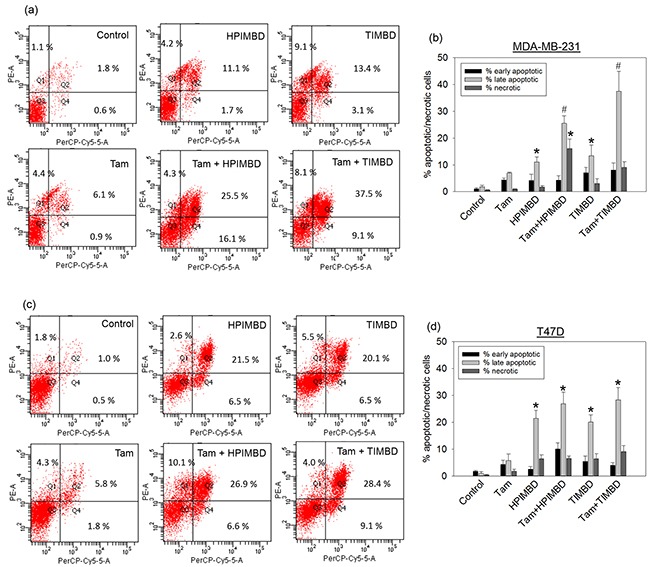
HPIMBD and TIMBD in combination with tamoxifen synergistically induce late-stage apoptosis in MDA-MB-231, but not in T47D breast cancer cells **a-b.** MDA-MB-231 cells were treated with either vehicle, 2 μM Tam, IC_50_ μM HPIMBD or TIMBD or a combination of Tam + HPIMBD or TIMBD for 48 hours and Annexin V/7-AAD staining was performed. Experiments were performed in triplicate and data are expressed as percentage of cells undergoing apoptosis or necrosis ± SEM. Representative scatter plots with % of cells in each quadrant are shown. (*) indicates a P value ≤ 0.05 compared to controls. (#) indicates a synergistic effect of a combination of Tam + HPIMBD/TIMBD compared to single drug treatments. **c-d.** T47D cells were treated with either vehicle, 2 μM Tam, IC_50_ μM HPIMBD or TIMBD or a combination of Tam + HPIMBD or TIMBD for 48 hours and Annexin V/7-AAD staining was performed. Experiments were performed in triplicate and data are expressed as percentage of cells undergoing apoptosis or necrosis ± SEM. Representative scatter plots with % of cells in each quadrant are shown. (*) indicates a P value ≤ 0.05 compared to controls.

Apoptosis and necrosis assays were performed to determine the mechanism of cell death induced by the combination treatment in breast cancer cells. T47D and MDA-MB-231 cells were treated with a combination of Tam (2μM) and HPIMBD or TIMBD (IC_50_ dose in respective cell line) for 48 hours and apoptosis/necrosis assays were performed by Annexin-V/7-AAD staining. Tamoxifen alone induced apoptosis in about 4% of cells, late-stage apoptosis in about 6% of cells and did not induce necrosis in both T47D and MDA-MB-231 cells after 48 hours of treatment (Figure 3a, b, c and 3d). HPIMBD and TIMBD significantly induced late apoptosis in about 11% and 13% of MDA-MB-231 cells respectively, which synergistically increased to 25% and 37% when treated with the combination of Tam and HPIMBD or TIMBD, respectively (Figure 3a and 3b). In T47D cells, both HPIMBD and TIMBD significantly induced late-stage apoptosis in 21% and 20% cells which further increased to 27% and 28% in cells treated with the combination, respectively (Figure 3c and 3d). HPIMBD and TIMBD induced more late-stage apoptosis in T47D cells compared to MDA-MB-231 cells to begin with, however, the combination treatment synergistically induced late apoptosis in MDA-MB-231 cells when compared to T47D cells (Figure 3a, b, c and 3d). This suggests that the combination of Tam and HPIMBD or TIMBD has a synergistic effect by the mechanism of inducing late-stage apoptosis in MDA-MB-231 cells but not as much in T47D cells.

### A combination of Tam with HPIMBD or TIMBD significantly induces autophagy markers beclin-1 and LC3BII in MDA-MB-231 breast cancer cells

**Figure 4:**
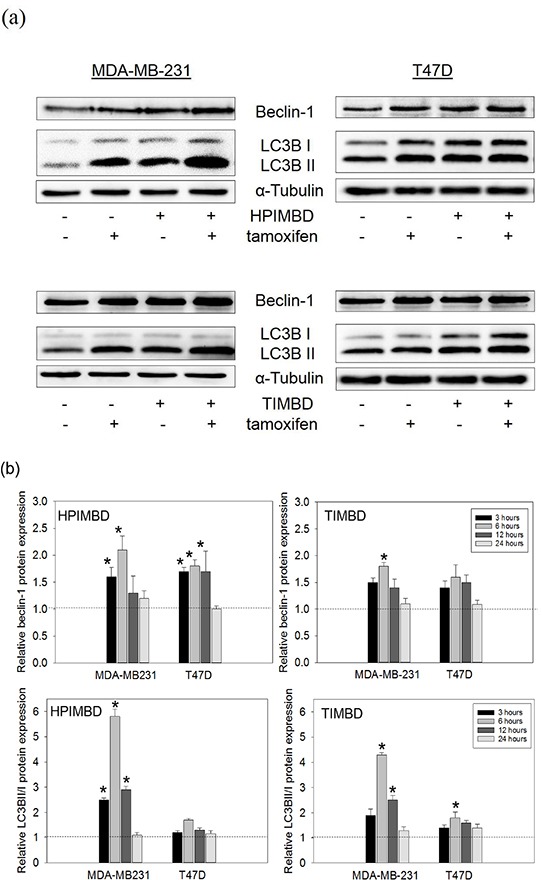
Tamoxifen in combination with HPIMBD or TIMBD, significantly induce autophagy markers beclin-1 and LC3BII in MDA-MB-231, but not in T47D breast cancer cells **a-b.** T47D and MDA-MB-231 cells were treated with either vehicle or 2 μM Tam, IC_50_ μM HPIMBD or TIMBD or a combination of Tam + HPIMBD or TIMBD for up to 24 hours and a time course study was performed. Proteins were isolated and western blot analyses were performed at each time point (3, 6, 12 and 24h). Intensities of the bands were quantified and normalized to α-tubulin. Fold changes in beclin-1 and ratio of LC3BII/I protein expression (Mean ± SEM) compared to vehicle-treated controls were calculated from three individual experiments and are expressed as bar graphs in fig. 4b. (*) indicates a P value ≤ 0.05 compared to controls of the same cell type. (#) indicates a synergistic effect of a combination of Tam + HPIMBD/TIMBD compared to single drug treatments.

We have previously shown that autophagy inducer, beclin-1 is increased in response to HPIMBD and TIMBD treatments [39]. We next assessed the expression of autophagy markers, beclin-1 and LC3BII, in response to the combination treatment. Breast cancer cells MDA-MB-231 and T47D were treated with a combination of Tam (2 μM) and HPIMBD or TIMBD (IC_50_ dose) for up to 24 hours. In MDA-MB-231 cells, the combination of Tam with both analogs synergistically induced the expressions of beclin-1 and LC3BII/I by more than 2-fold and 5-fold, respectively after 6 hours of treatment (Figure 4a and 4b). In T47D cells, however, the induction of both beclin-1 and LC3BII were not synergistic even after 24 hours of treatment (Figure 4a and 4b). A time-course study showed that the combination significantly induced both beclin-1 and LC3BII/I in MDA-MB-231 cells after 3, 6 and 12 hours, with maximal induction observed at 6 hours post treatment (Figure 4b). HPIMBD was better than TIMBD in inducing both beclin-1 and LC3BII/I in MDA-MB-231 cells (Figure 4a and 4b). Our studies show a cell-type specific mechanism of synergy in inhibition of cancer cells by the combination; with apoptosis and autophagy being synergistically induced only in ER-negative MDA-MB-231 cells.

### A combination of Tam with HPIMBD or TIMBD significantly inhibited protein expression levels of ERα and c-Myc in MCF-7 and T47D breast cancer cells

**Figure 5:**
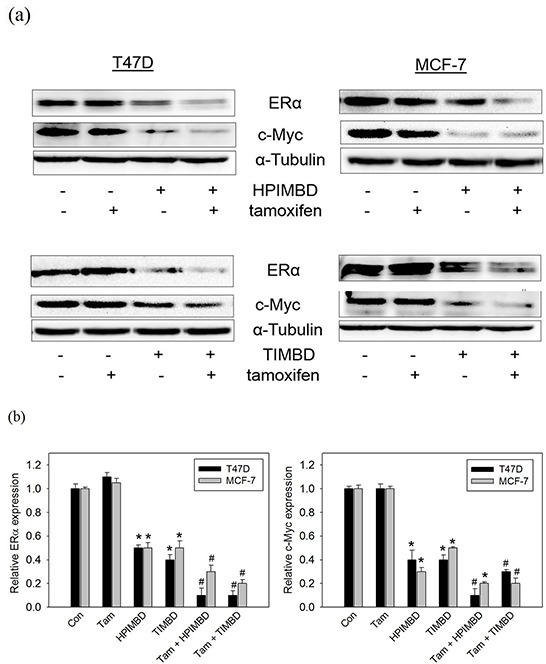
Tamoxifen in combination with HPIMBD or TIMBD, significantly inhibited the protein expressions of ERα and c-Myc in MCF-7 and T47D breast cancer cells **a-b.** MCF-7 and T47D cells were treated with either vehicle or 2 μM Tam, IC_50_ μM HPIMBD or TIMBD or a combination of Tam + HPIMBD or TIMBD for 48 hours. Proteins were isolated and western blot analyses were performed for ERα and c-Myc. Intensities of the bands were quantified and normalized to α-tubulin. Fold changes in ERα and c-Myc protein expression (Mean ± SEM) compared to vehicle-treated controls were calculated from three individual experiments and are expressed as bar graphs in figure 5b. (*) indicates a P value ≤ 0.05 compared to controls of the same cell type. (#) indicates a synergistic effect of a combination of Tam + HPIMBD/TIMBD compared to single drug treatments.

We have previously shown that both HPIMBD and TIMBD significantly inhibit protein expression levels of ERα and oncogene c-Myc in breast cancer cell lines MCF-7 and T47D [40, 41]. We investigated the effect of the combination on ERα and c-Myc expression. Breast cancer cells MCF-7 and T47D were treated with a combination of Tam (2μM) and HPIMBD or TIMBD (IC_50_ dose) for 48 hours and protein expression levels of ERα were assessed. Tamoxifen by itself did not change the expression of ERα in cells (Figure 5a and 5b). Both HPIMBD and TIMBD alone inhibited the expression of ERα by 50-60%, which was synergistically inhibited by the combination up to 70-90% (Figure 5b). Both HPIMBD and TIMBD alone inhibited c-Myc expression by 50-70%, which was further synergistically inhibited by the combination up to 70-90% (Figure 5b). This suggested that in ERα-positive cell lines, inhibition of ERα and its downstream oncogene c-Myc may be the mechanism of synergistic inhibition of breast cancer cells.

### A combination of Tam with HPIMBD or TIMBD inhibited the spheroid forming and colony forming ability of breast cancer cells

**Figure 6:**
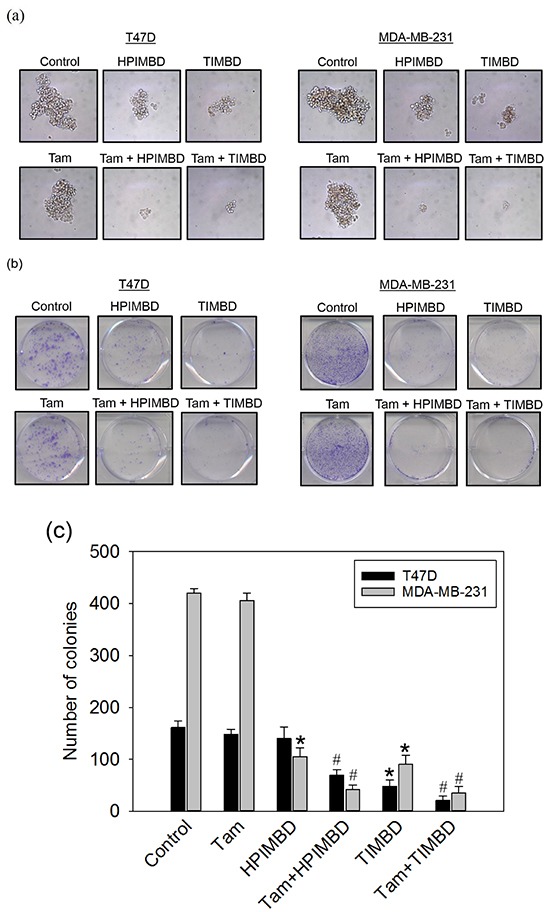
Tamoxifen in combination with HPIMBD or TIMBD, inhibited the spheroid forming and colony forming ability of MDA-MB-231 and T47D breast cancer cells **a.** T47D and MDA-MB-231 breast cancer cells were seeded in suspension in ultra-low attachment plates followed by treatments with 2 μM Tam, IC_50_ μM HPIMBD or TIMBD or a combination of Tam + HPIMBD or TIMBD. After 5 days of incubation, mammospheres formed in suspension were photographed and representative photomicrographs from triplicates are shown. **b-c.** T47D or MDA-MB-231 cells were seeded in triplicate in six-well plates followed by treatments with 2 μM Tam, IC_50_ μM HPIMBD or TIMBD or a combination of Tam + HPIMBD or TIMBD for 48 hours. Treatment medium was replaced with fresh media and cells were allowed to form colonies for 8 days. Colonies obtained were stained with crystal violet, photographed and counted (b). The bar graph (c) represents mean number of colonies ± SEM from three experiments. (*) indicates a P value ≤ 0.05 compared to controls of the same cell type. (#) indicates a synergistic effect of a combination of Tam + HPIMBD/TIMBD compared to single drug treatments.

The effect of a combination of Tam with Res analogs on cancer cell proliferation was evaluated by performing spheroid formation and colony formation assays. Tamoxifen treatment alone only slightly suppressed the size of spheroids and number of colonies in both T47D and MDA-MB-231 cells (Figure 6a and 6b). HPIMBD and TIMBD treatment substantially inhibited the colony forming and spheroid forming ability of T47D and MDA-MB-231 cells, which was further inhibited by the combination treatment (Figure 6a and 6b). Statistical analyses of colony formation studies revealed synergistic inhibitory effect of the combination in both the breast cancer cell lines tested (Figure 6c).

## DISCUSSION

Tamoxifen is not only the first line of therapy for women affected by breast cancer but is also most often used in combination therapies [16, 17, 28–31]. Tamoxifen is also widely used in chemoprevention and clinical studies have shown that Tam can significantly reduce cancer occurrence and improve survival among women with high risk for breast cancer [42, 43]. Combination therapies have many advantages over single drug treatments [28–31]. Due to the increased toxicity with synthetic agents, alternative combinations with agents of natural origin are being tested [29–31]. Furanodiene, a natural component of Curcuma Wenyujin plant, has been reported to increase the anti-cancer efficacy of Tam in inhibiting the growth of ER-negative MDA-MB-231 cells, broadening its use to hormone-independent types of breast cancers [29]. Curcumin, a natural component of turmeric, restores the sensitivity of resistant MCF-7 cells to Tam [31]. Epigallocatechin gallate, a component of green tea, synergistically inhibits the growth of ER-negative MDA-MB-231 cells in combination with Tam [30].

In the current study, the combination of Tam and Res analogs HPIMBD or TIMBD were evaluated. A low dose of Tam (2 μM) was chosen such that it does not inhibit the proliferation of non-neoplastic breast epithelial cells. The combination of Tam with HPIMBD or TIMBD did not inhibit the growth of non-neoplastic breast epithelial cells, suggesting least cytotoxicity to normal cells (Figure 1b). When tested on breast cancer cell lines, the combination treatment synergistically inhibited proliferation compared to either drug treatments alone (Figure 2a and Table 1). Dose-response curves plotted with varying doses of HPIMBD or TIMBD in combination with constant low dose of Tam showed that the combination had significantly lower IC_50_ compared to that of HPIMBD or TIMBD treatments alone (Table 2). Mechanistic studies showed that the mechanism of synergistic inhibition in breast cancer cells correlated with their receptor status. In ER-negative MDA-MB-231 cells, the combination synergistically induced apoptosis and autophagy markers, while in ER-positive T47D cells, synergistic inhibition of ERα and c-Myc protein expression was observed (Figures 3, 4 and 5). These studies suggest that, in ER-positive cells, synergistic inhibition may occur in an ER-dependent fashion while in ER-negative cells, the key upstream regulatory pathway operating the effect may be early induction of autophagy followed by late-stage apoptosis. Interestingly, literature provides evidence that autophagy precedes apoptosis, and may act as an initial response to the drug/agent and induce late-stage apoptosis in cells [44, 45]. Since we observed a similar effect of the combination on MDA-MB-231 cells, we hypothesize that there may be a link between the two pathways induced by the combination in breast cancer cells. Colony and spheroid formation assays further confirmed the synergistic inhibition of breast cancer cells by the combination (Figure 6).

One of the problems associated with the use of Tam in chemoprevention is low adherence to therapy among women which is attributed to result from increased adverse/toxic effects [46, 47]. Reduced toxicity of our combination may thus have a potential of increasing adherence and advancing treatment options for chemoprevention. Current management for women with advanced and metastatic breast cancer include surgery with chemotherapy and/or radiation therapy followed by long term targeted therapy [48]. Five years of tamoxifen is the standard of care for ER-positive cancers, trastuzumab for HER2+ cancers while the therapy for triple-negative breast cancers is not targeted [48, 49]. We have shown that Tam combined with Res analogs can inhibit the growth of both ER-positive and ER-negative breast cancer cells, creating the potential for extending the use of Tam to triple negative breast cancers that are otherwise difficult to treat [49]. Severe adverse effects and relapses associated with single drug Tam therapy have led to the need for newer well-tolerable drugs that alone or in combination with Tam can help in better management and enhanced quality of life for patients [24–27]. Aromatase inhibitors and fulvestrant are the currently approved alternative drugs for anti-hormonal therapy [50]. Of particular interest is fulvestrant which has been reported to have good efficacy and acts by binding to ERα and causing its degradation, thus inhibiting receptor expression [50]. HPIMBD and TIMBD also inhibit the expression of ERα in breast cancer cells and this similarity in mechanism along with low toxicity make the novel Res analogs attractive drug candidates for translational investigations.

In summary, we have, for the first time, shown that a combination of low dose Tam with novel azares analogs can synergistically inhibit the proliferation of both ER-positive and ER-negative breast cancer cells. The combination, having reduced toxicity to normal breast epithelial cells, has a potential in both chemoprevention and clinical therapy.

## MATERIALS AND METHODS

### Chemicals

Tamoxifen was purchased from Tocris (Minneapolis, MN). Resveratrol was purchased from Sigma-Aldrich (St. Louis, MO). Resveratrol analogs, HPIMBD and TIMBD were synthesized and purified by our group as reported recently [39]. Tamoxifen, HPIMBD and TIMBD were dissolved in DMSO prior to treatments. The concentration of DMSO in control experiments was always 1/1000^th^ (vol/vol) of the final medium volume. 3-(4,5-Dimethyl-2-thiazolyl)-2,5-diphenyl-2H-tetrazolium bromide (MTT) was purchased from Sigma-Aldrich (St. Louis, MO). MTT reagent was prepared by dissolving MTT in PBS to a final concentration of 1mg/ml which was used as a stock solution.

### Cell culture

Non-tumorigenic breast epithelial cell lines MCF-10A, MCF-10F and HMEC, and breast cancer cell lines MCF-7, T47D, MDA-MB-231 and MDA-MB-468 were purchased from ATCC (Manassas, VA). Breast epithelial cell lines MCF-10A, MCF-10F and HMEC were cultured in DMEM/F-12 (50:50) media (Mediatech, Herndon VA) that was supplemented with 10% horse serum (Atlanta Biologicals, Lawrenceville GA) and 1% penicillin/streptomycin antibiotic (Lonza). Breast cancer cell lines MCF-7, T47D, MDA-MB-231 and MDA-MB-468 were cultured in DMEM/F-12 (50:50) media (Mediatech, Herndon VA) that was supplemented with 10% fetal bovine serum (Atlanta Biologicals, Lawrenceville GA) and 1% penicillin/streptomycin antibiotic (Lonza). Cells from respective cell lines were seeded in 6-well or 96-well tissue culture plates and were grown till they achieved ≃70% confluency. Twenty-four hours prior to treatments, cells were washed twice with PBS and then grown in phenol red-free DMEM/F-12 (50:50) supplemented with 10% charcoal dextran stripped fetal bovine serum or horse serum for respective cell lines (Atlanta Biologicals, Lawrenceville GA). Next day, cells were treated with either HPIMBD or TIMBD (at respective IC_50_ doses for each cell line) or Tam (variable doses) or a combination of Tam + HPIMBD or TIMBD for up to 72 hours. All treatments were done in triplicates or quadruplicates. Experiments were performed in passages 2 to 6 of cells sub-cultured from frozen stocks of respective cell lines. After treatments, the media was removed and cells were washed once with PBS and were used for further analyses.

### MTT cell proliferation assays

The effect of a combination of HPIMBD or TIMBD and Tam on the viability of breast cancer cells was measured by MTT assays as described recently [39, 40, 41]. Cells were plated at a density of 2000 cells/well into 96-well plates. After overnight incubation in respective growth media, cells were treated with either HPIMBD or TIMBD (at respective IC_50_ concentration for each breast cancer cell line and 50μM concentration in non-neoplastic cell lines) or Tam (2μM) or a combination of Tam + HPIMBD or TIMBD. After 72 hours, 50μl of MTT reagent was added to each well and incubated for 4 hours at 37°C. The medium was then removed and 200μl of a mixture of DMSO and Sorensen's reagent (volume ratio 8:1) was added. The plate was then incubated at 37°C for 10 mins and the optical density for each well was determined at 570 and 650 nm on BIO-RAD Benchmark Plus microplate spectrophotometer (Biorad, Hercules, CA) using the Microplate Manager 5.2.1 software. The absorbance at 650 nm was subtracted from absorbance at 570 nm. This value was then used to calculate mean % cell viability compared to respective vehicle-treated controls. Percent inhibition of proliferation were calculated by subtracting % cell viability from 100. Percent inhibition of proliferation compared to controls are expressed as mean ± SEM.

### Calculation of IC_50_ values of the combinations in breast cancer cell lines

To determine the IC_50_ values of a combination of Tam + HPIMBD or TIMBD, cells were treated with a combination of fixed dose of Tam (2 μM) with increasing doses of either HPIMBD or TIMBD and MTT assays were performed for 72 hours. Each experiment was performed in triplicate. Sigmoidal curves for each replicate values were plotted using Regression Wizard in Sigma Plot 11.0 software (Systat Software Incorporated, CA) and the four parameter logistic (4PL) mathematical model was used to obtain equations for Tam + HPIMBD or TIMBD in each of the four breast cancer cell lines tested [51, 52]. The 4PL formula used for IC_50_ calculation is expressed as:

$$\text{y}={\text{y}}_{0}+\frac{\text{a}}{1+{\left(\text{x}/{\text{x}}_{0}\right)}^{\text{b}}}$$

Where y is fold change in proliferation relative to respective vehicle control of each cell line and x is dose/concentration of HPIMBD/TIMBD in the combination, while a, b, x_0_ and y_0_ are constants obtained by non-linear regression of the curve. Corresponding IC_50_ values of were obtained by substituting y as 0.50 in each equation generated from three replicate curves for a given cell line and analog in combination. In this way, three IC_50_ values were obtained and averaged for each combination (Tam + HPIMBD or TIMBD) in each of the four tested cell lines. Average IC_50_ μM ± SEM are listed in Table 2.

### Apoptosis and necrosis assays

Breast cancer cells were plated in 6-well plates and were treated with either HPIMBD, TIMBD, Tam or a combination of Tam + HPIMBD or TIMBD for 48 hours. Post-treatment, apoptosis and necrosis analyses were performed using PE Annexin V Apoptosis Detection Kit I from BD Pharmingen^TM^(BD Biosciences, San Jose, CA). Briefly, after completion of treatments, cells were washed twice with cold PBS and were resuspended at a concentration of 1 × 10^6^ cells/ml. Annexin V and 7-AAD were added at concentrations specified in the kit and were incubated for 15 minutes at room temperature in the dark. After incubation, % of apoptotic and necrotic cells was analyzed by flow cytometry using a BD FACSCanto^TM^ II cell analyzer (BD Biosciences, San Jose, CA, USA). The data were acquired and analyzed with BD FACSDiva software.

### Western blot analysis

Thirty microgram total protein, isolated from quadruplicates of control or treated cells, was size fractionated on a 12% SDS-polyacrylamide gel, and transferred onto PVDF membranes under standard conditions as described previously [53–57]. Membranes were blocked in 5% dry non-fat milk/PBS/0.05% Tween-20 at four degrees for two hours. Affinity purified rabbit polyclonal antibodies against beclin-1 (Cell Signaling, 3738S), LC3B (Cell Signaling, 2775S), ERα (Santa Cruz, sc-543) and c-Myc (Santa Cruz, sc-788) were used for immune-detection. After overnight incubation with the primary antibodies in cold cabinets, membranes were washed three times for 8 minutes per wash using PBS/0.05% Tween-20. Horse radish peroxidase conjugated anti-rabbit IgG (Santa Cruz, sc-2004) was used as secondary antibody. After incubation for one hour at room temperature, the membrane was washed again as described above. Chemiluminescent detection was performed using the BM Chemiluminescence Detection kit (Roche, Indianapolis, IN) and the FluorChem HD2 Imaging system (Alpha Innotech Corporation, San Leandro CA), with AlphaEaseFC Image Analysis software (Alpha Innotech Corporation, San Leandro CA). Membranes probed for beclin-1, LC3B, ERα and c-Myc were washed twice in PBS/0.05% Tween-20, stripped with Restore Western blot stripping buffer (Thermo Scientific, Rockford, IL), blocked in milk and re-incubated overnight at room temperature with α-tubulin mouse monoclonal antibody (Santa Cruz, sc-53030). Horse radish peroxidase-conjugated anti-rat IgG antibody (Santa Cruz, sc-2006) was used as secondary antibody for α-tubulin detection. All primary and secondary antibodies were diluted to 1:2000 concentration in PBS/0.05% Tween-20 prior to immune detection.

### Clonogenic cell survival assay

About 500 viable MDA-MB-231 and T47D cells were seeded in 6-well plates. Next day, the media was changed to phenol red-free medium and cells were allowed to grow for another 24 hours. Cells were then treated with either HPIMBD, TIMBD, Tam or a combination of Tam + HPIMBD or TIMBD for 48 hours after which they were washed in PBS and incubated for an additional 8 days in complete medium. The colonies obtained were washed with PBS and fixed in 10% formalin for 10 min and again washed twice with PBS followed by staining with crystal violet (0.1% w/v solution in 10% ethanol). The colonies were counted, photographed and compared with respective untreated cells as described previously [58–60]. Each treatment was done in triplicate.

### Mammosphere formation assay

Mammosphere formation assay was carried out using ultra-low attachment plates (catalog # 3473; Corning, Lowell, MA) as described previously [58–60]. Briefly, 5000 viable MDA-MB-231 or T47D cancer cells were seeded into 24-well plates. Cells were grown in serum-free and phenol red-free DMEM/F-12 (50:50) medium supplemented with 1×B27 (Invitrogen), 20 ng/ml epidermal growth factor (Invitrogen), 20 ng/ml basic fibroblast growth factor (Invitrogen), 1 μg/ml hydrocortisone (BD Biosciences, Bedford, MA), 5 μg/ml insulin (Invitrogen), 0.1% penicillin/streptomycin (Lonza, Walkersville, MD) and 4 μg/ml heparin calcium salt (Thermo Scientific) at 37°C under 5% CO_2_. Cells were treated with HPIMBD, TIMBD, Tam, Tam + HPIMBD or Tam + TIMBD. After 4 days of incubation, mammospheres were viewed under the microscope and photographed. Three replicate wells from a 24-well plate were used for each experimental condition.

### Statistical analysis

Statistical analyses were performed by using Sigma Plot 11.0 software (Systat Software Incorporated, CA). All cell culture treatments were done in triplicate or quadruplicate and the student's t-test was used to compare the inhibition in proliferation by either Tam, HPIMBD, TIMBD or a combination of Tam and HPIMBD or TIMBD compared to respective vehicle treated controls. In order to determine if the combinations resulted in a synergistic effect compared to either treatments alone, two-way ANOVA analyses were performed in a generalized linear model using the IBM SPSS Statistics Data Editor software (IBM Corporation, Armonk, NY). A p-value ≤ 0.05 was considered statistically significant.
